# Organization of a Medical Society, Keokuk

**Published:** 1856-04

**Authors:** 


### ORGANIZATION OF A MEDICAL SOCIETY.

It will be seen by the following proceedings, furnished us by the Secretary, that a Medical Society has been organized in this city, for the purposes as set forth, of scientific improvement and the advancement of the interests of the profession, not of this city only, but of the State and those portions of States lying contiguous.

The design of the founders is to place it upon such a basis as to ensure its permanency, and at the same time, to place it upon an elevated footing. None other than graduates of a respectable medical institution will be admitted, and these must, in addition to scientific qualifications, present themselves without "spot and blemish" so far as the moral deportment, and the professional standing of the applicant among their brethren, are concerned. Those who treat the rules of medical etiquette with contempt, and their professional brethren in a manner unbecoming the high character which the profession should maintain for probity and honor, will be rejected. The constitution provides for two classes of members, *active* and *privileged*, the former to compose the working members, while the latter may reside at a distance. These, when they apply for membership, will furnish indubitable evidence of the possession of a *diploma* of a respectable regular medical institution, and with it the proofs of a good moral standing and private character. None else need apply. It is the intention to make it such an organization as the best medical men of the country will find it pleasant and profitable to become members of.

Papers will be read at each meeting, and these papers may be presented by the *active* in person, or may be forwarded by *privileged* members, through the mail, to the Secretary.

Discussions on medical topics will be had, and important facts in medical literature, and the new discoveries in science will be presented.

A neat certificate of membership will be prepared and issued to each member. Already several gentlemen living in the surrounding country have intimated their desire to become members.

The result of this organization will be most favorable to the profession. It will tend to elevate it, by requiring the usual evidences of qualification, which all may secure at this day, if they but seize hold of the benefits now so easily secured, of completing their education. The Constitution and By-Laws will be printed for distribution, and appear also in the pages of our next issue.

THE DUDLEYAN MEDICAL SOCIETY OF KEOKUK, IOWA. Apr(?) 19, 1856

At a meeting of the Physicians of Keokuk, held in accordance with previous notice, on the 19th inst., at the office of Drs. M'Gugin and Allen, on motion, Dr. Knowles was called to the Chair, and Dr. Marsh appointed Secretary pro tem. Dr. M'Gugin then stated the object of the meeting to be the formation of a city society, for the purpose of advancing professional and scientific knowledge, and the cultivation of social and friendly relations among its members.

After discussing the plan to be pursued in the proposed organization, a committee of three was appointed to draft a Constitution and By-Laws.

After a recess, the committee reported a Constitution and By-Laws, which were unanimously adopted.

Officers were then duly elected, as prescribed by the Constitution.

*President*—D. L. M'Gugin, M. D.

*Vice President*—Freeman Knowles, M. D.

*Recording Secretary*—L. Wood, M. D.

*Corresponding Secretary*—Wells R. Marsh, M. D.

*Treasurer*—J. J. Page, M. D.

The society then adjourned to meet again on the first Monday of May next, at the office of Drs. M'Gugin and Allen.

D. L. M'GUGIN, M. D., *President.*

L. Wood, M. D., *Secretary.*

